# Microbiota-Gut-Kidney Axis and Targeted Therapeutic Strategies in Kidney Diseases

**DOI:** 10.7150/ijbs.124499

**Published:** 2026-01-01

**Authors:** Zheng-hao Sun, Qian Gong, Zi-lai Wang, Chao Li, Jia-nan Wang, Ju-tao Yu, Ming-lu Ji, Dan-feng Zhang, Jie Wei, Shuai-shuai Xie, Fang-jie Zhou, Xiang-yu Li, Xiao-guo Suo, Qi Zhu, Juan Jin, Wei Shao, Pei Pan, Xiao-ming Meng

**Affiliations:** 1 School of Basic Medical Sciences, Anhui Medical University, Hefei 230032, China.; 2 Department of Cardiovascular Surgery, The First Affiliated Hospital of Anhui Medical University, Hefei 230032, China.; 3 Inflammation and Immune Mediated Diseases Laboratory of Anhui Province, The Key Laboratory of Anti-Inflammatory of Immune Medicines, Ministry of Education, Anhui Institute of Innovative Drugs, School of Pharmacy, Anhui Medical University, Hefei 230032, China.; 4 Department of Nephrology, the Second Affiliated Hospital of Anhui Medical University, 678 Furong Road, Hefei 230601, Anhui, China.

**Keywords:** microbiota-gut-kidney axis, kidney diseases, therapeutic strategies, gut microbiota dysbiosis

## Abstract

The gut microbiota, as a source of profound genetic and metabolic capacity, affects every aspect of human biology including health, development and aging, as well as disease. Studies have demonstrated the crosstalk between the gut microbiota and the kidney, which is directly referred to as the “microbiota-gut-kidney axis”. Most gut microbiota metabolites are associated with metabolic, immune, and inflammatory pathways. Disruption of gut microbiota homeostasis in patients with kidney disease contributes to further loss of kidney function, forming a vicious cycle. This discovery may provide a potential avenue of treatment for kidney diseases, creating a new therapeutic paradigm. Moreover, new therapeutic strategies seem to be beneficial for kidney health via the modulation of the gut microbiota. Although these strategies show early beneficial results, their long-term effectiveness and safety require further investigation and confirmation. In this review, we discuss the interactions between the gut microbiota and kidney diseases and explore related therapeutic strategies. A comprehensive understanding of the microbiota-gut-kidney axis will facilitate the development of efficient therapeutic measures for kidney diseases.

## 1. Introduction

Kidney diseases, including acute kidney injury (AKI), chronic kidney disease (CKD), renal cell carcinoma (RCC) and various renal dysfunctions have emerged as crucial public health issues globally 1. They are highly prevalent, with incidence rates continuing to increase, and are strongly linked to metabolic disorders and immune dysfunction 1-3. The kidneys, one of the organs with the highest metabolic rates in the human body, play an irreplaceable role in maintaining internal body fluid homeostasis by filtering approximately 180 liters of plasma (primary urine) each day 2. In addition to waste excretion, the kidneys are crucial for regulating blood pressure, electrolyte balance, and certain endocrine functions 2. Recently, an increasing number of researchers have pointed out the crosstalk between the kidneys and other organs 4-6, suggesting that kidney diseases may affect multiple organs through complex pathways.

In recent years, the microbiota-gut-kidney axis has received increased attention. Trillions of bacteria inhabit the gut, which is the largest microecosystem, and alterations in gut bacteria influence host health 7. Given the function of the gut microbiota in immune homeostasis modulation, intestinal epithelial barrier integrity maintenance, and metabolite generation, the gut microbiota displays potent systemic roles in other non-intestinal organs, including the kidneys 8-10. Diseases such as diabetes, obesity, and inflammatory bowel disease are strongly associated with gut microbiota dysbiosis 11-13. The relationship between gut microbiota and kidney diseases has received considerable attention. In patients with kidney disease, gut microbiota dysbiosis manifests as relatively low diversity and a higher abundance of pathogenic bacteria, which might lead to systemic inflammation and kidney injury 14. Furthermore, kidney disease is associated with alterations in microbiota-derived metabolites that are thought to be critical for disease development. For example, the accumulation of uremic toxins in patients with CKD and patients undergoing dialysis can lead to kidney injury 15, 16.

Given the close relationship between gut microbiota and kidney diseases, strategies targeting the gut microbiota have become a focus of research. For example, Modified Huangfeng Decoction has been shown to ameliorate diabetic kidney disease (DKD) by modulating gut microbiota and its metabolites 17. *Lactobacillus reuteri* can influence the gut microbiota, leading to reduced glycated hemoglobin and serum cholesterol in type 2 diabetes (T2D) patients 18. Administration of the probiotic *Lactobacillus casei Zhang* to CKD patients can slow the decline in kidney function 19. As research progresses, these strategies are increasingly demonstrating clinical translational potential. In this review, we provide a comprehensive overview of the microbiota-gut-kidney axis and its potential therapeutic targets and modulations, such as probiotics, synbiotics, fecal microbiota transplantation (FMT), and dietary intake, as well as the potential use of this new burgeoning field for the treatment of kidney diseases. This will improve understanding of the pathways of molecular mechanisms of kidney diseases and the development of new drugs targeting the microbiota-gut-kidney axis.

## 2. Microbiota-gut-kidney axis in kidney diseases

### 2.1 Microbiota-gut-kidney axis

**Figure 1:**
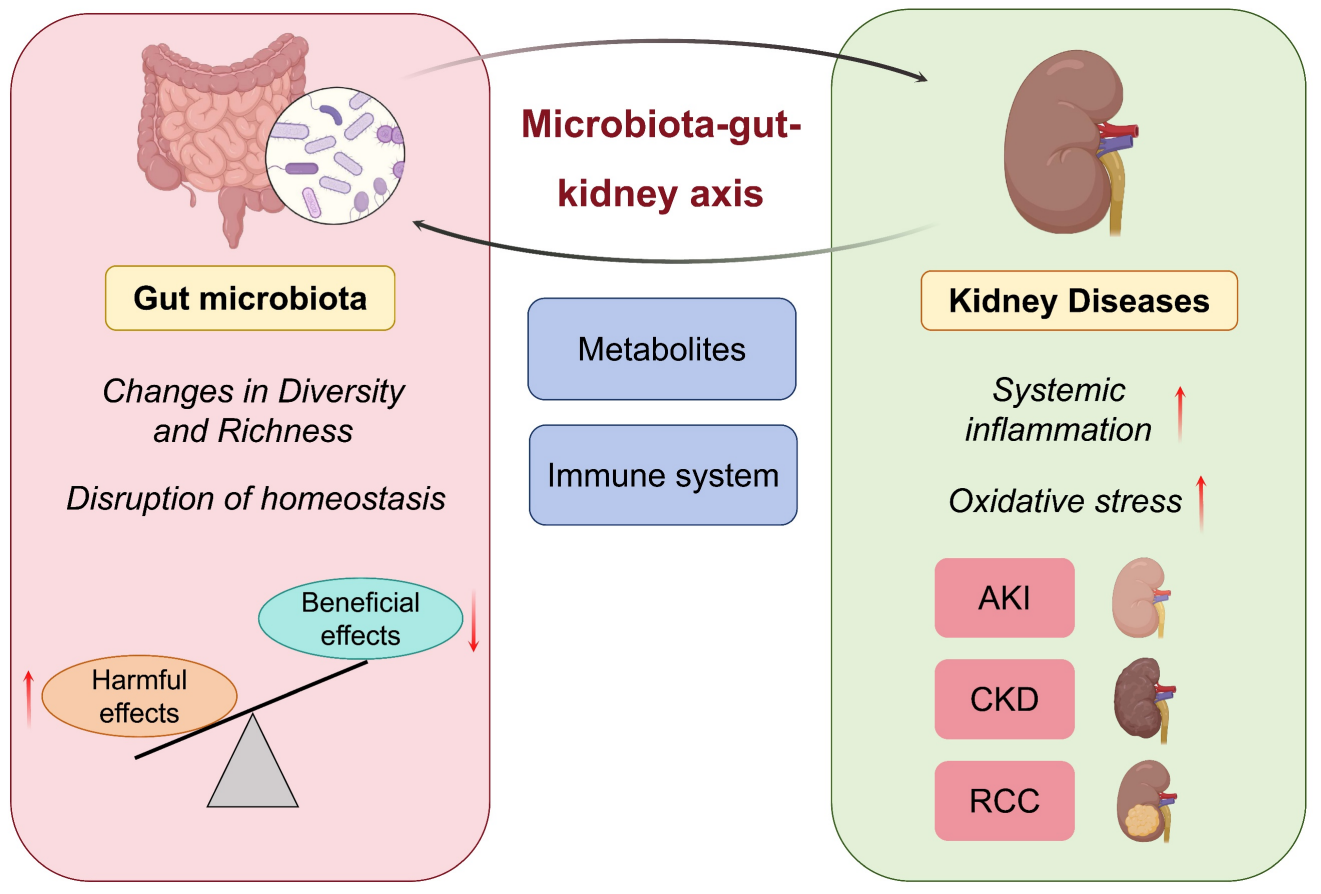
Microbiota-gut-kidney axis in kidney diseases. Gut microbiota exhibits bidirectional interplay with various kidney diseases.

The microbiota-gut-kidney axis is a new research field that has attracted more and more attention from scholars. The relationship between gut microbiota and kidney diseases has emerged as a novel concept in nephrology research. This may overthrow the traditional concept of kidney disease and lay the foundation for new theories and clinical guidelines for the prevention and treatment of kidney diseases. The gut microbiota affects kidney function via metabolic and immunological pathways, and kidney disease can further shape the gut microbiota by disturbing systemic metabolism and immune responses 19, 20. One study demonstrated that patients with kidney disease exhibited reduced gut microbiota richness and an increased proportion of pathogenic species. Harmful bacteria generate metabolites that induce harmful pathways of inflammation and oxidative stress, ultimately leading to the worsening of kidney function 21. When kidney function declines to levels at which metabolic waste is no longer adequately filtered from the bloodstream, gut microbiota dysbiosis is promoted, creating a vicious cycle (Figure 1). To combat this, researchers have scrambled to develop strategies to escape this spiral. Evidence has shown that rehabilitation of the gut microbial environment mitigates the progression of kidney diseases. It has been suggested that probiotics, which have the potential to reduce the production of uremic toxins by restoring gut microbiota balance, may improve clinical outcomes in CKD patients 22. Another study suggested that FMT could restore gut microbiota homeostasis and intestinal integrity to reduce the burden on the kidneys 23. These findings demonstrate the therapeutic potential of regulating the gut microbiota in kidney disease management.

### 2.2 Gut microbiota-derived metabolites in kidney diseases

Metabolites are directly influenced by compositional changes in the gut microbiota and are closely associated with the progression of kidney disease 24. Gut microbiota dysbiosis promotes the production of harmful metabolites and impairs the intestinal barrier, leading to an increased absorption of toxic substances. This process can exacerbate kidney inflammation and injury 25. During the course of kidney disease, various dangerous metabolites, such as indoxyl sulfate (IS), p-cresol sulfate (PCS), trimethylamine N-oxide (TMAO), and Advanced Glycation End Products (AGEs), are produced.

With decreased kidney function, IS and PCS, which are toxic metabolites produced by the gut microbiota, freely accumulate in the plasma, leading to deleterious health consequences. IS is a tryptophan metabolite that is naturally excreted in urine by the kidneys. IS accumulates in the circulation due to kidney dysfunction, contributing to oxidative stress and inflammation, which promotes the progression of kidney fibrosis 26. It has been reported that higher IS levels not only inhibit the growth of beneficial bacteria including *Bifidobacterium* and *Lactobacillus*, but also increase the ratio of pathogenic bacteria, and therefore aggravate systemic inflammation 27. PCS induces the production of reactive oxygen species (ROS), leading to oxidative stress and promoting the progression of kidney disease 28. Furthermore, PCS can induce insulin resistance in CKD, and blocking PCS may be a potential therapeutic approach for the treatment of CKD 29. TMAO is another common harmful metabolite. The gut microbiota generates TMA during the metabolism of choline and L-carnitine, and TMA is oxidized by the liver to TMAO. An imbalance in the gut microbiota is closely associated with the development and prognosis of CKD, and increased TMAO level is an important factor in its prognosis. These findings suggest that interventions to prevent TMAO elevation may be beneficial in the prevention and prognosis of kidney disease. For example, inhibiting TMAO generation significantly alleviated kidney injury in adenine-induced CKD mice 30, 31. AGEs are noxious and permanent products of non-enzymatic reactions between carbonyl compounds and proteins or lipids. AGEs may disrupt the structure and function of the kidney through accumulation and induce downstream signaling of the AGE-receptor for AGEs pathway, thereby initiating oxidative stress and a pronounced inflammatory process, leading to further kidney injury 32. In summary, gut microbiota-derived metabolites are essential mediators of the microbiome-gut-kidney axis, and their homeostasis may be clinically relevant in the prevention and treatment of kidney diseases.

### 2.3 Interaction between gut microbiota and AKI

#### 2.3.1 Impact of AKI on gut microbiota

AKI is a clinical syndrome characterized by a rapid decline in kidney function and diverse pathogenic mechanisms. The gut microbiota confers several beneficial effects on the host by preserving intestinal barrier function, modulating the immune system, and producing vital metabolites such as short chain fatty acids (SCFAs). Nevertheless, in AKI, kidney injury may perturb this balance resulting in gut microbiota dysbiosis. In a cisplatin-induced AKI model, a reduction in the beneficial bacterium* Lactobacillus* was observed in feces 33. In a rat renal ischemia/reperfusion (I/R) AKI model, the levels of *Staphylococcus spp.* and *Rothia spp.* were increased and were positively associated with increased serum levels of creatinine and urea 34. AKI disrupts gut microbiota homeostasis via various mechanisms. First, AKI alters the anatomy and physiology of the gut mucosa. AKI-induced systemic inflammation and metabolic abnormalities affect the intestinal microenvironment, which is characterized by intestinal inflammation, damage, increased permeability, and altered mucus secretion. Such alterations alter the living environment of the gut microbiota, further altering its composition and structure 35, 36. Moreover, impaired kidney excretory function for uremic toxins and metabolic waste products in AKI can cause the retention of these substances, some of which can translocate to the intestinal tract via systemic circulation, thereby affecting the growth and metabolic activity of the gut microbiota. These alterations manifest as a decrease in beneficial bacteria and an increase in pathogenic bacteria 36-38. In addition, since AKI causes immune dysregulation, homeostasis of the gut microbiota could be disturbed. The pro-inflammatory cytokines and mediators released by the activated immune system can affect gut microbiota composition 38. For example, AKI has been found to disturb intestinal immune homeostasis, which is characterized by increased Th1 and Th17 responses, higher infiltration of neutrophils and pro-inflammatory M1 macrophages, as well as upregulated levels of IFN-γ 38. These changes not only affect the function of the intestinal barrier, resulting in a greater inflammatory response, but also enable the entry of gut-derived toxins into the blood circulation, aggravating kidney injury.

Fortunately, researchers have identified several therapies that can alleviate AKI dysbiosis of the gut microbiota. For example, modulation of gut microbiota through probiotics, prebiotics, or supplementation with SCFAs may help attenuate inflammation and kidney injury in AKI 19, 39. The second case involves Qiongyu paste, a traditional Chinese medicine product that modulates the gut microbiota and increases the production of SCFAs to attenuate kidney fibrosis and inflammation in AKI 40. In summary, AKI induces an imbalance in the gut microbiota, which exacerbates kidney injury through a vicious cycle.

#### 2.3.2 Involvement of gut microbiota in AKI

In contrast, disrupted gut microbiota has emerged as a crucial contributor to the development of AKI. Transplantation of the gut microbiota from AKI mice into germ-free mice results in exacerbated kidney injury 41. Consequently, researchers have proposed that modulation of gut microbiota dysbiosis could serve as an innovative therapeutic approach for the treatment of AKI. For instance, the administration of antibiotics to mice to deplete the gut microbiota attenuated the recruitment of local renal macrophages and bone marrow monocytes, impaired inflammatory responses, and protected against tubular injury of the kidneys 42. In mouse kidneys, depletion of gut microbiota with a cocktail of antibiotics before renal I/R injury could significantly reduce the concentrations of TNF-α, IL-6, and MCP-1 42.

Alterations in the composition of the gut microbiota have been linked to significant modifications in metabolite profiles, with recent research highlighting the significance of gut microbiota-derived metabolites in AKI (Table 1). Notably, SCFAs and amino acid metabolites play pivotal roles in immune regulation and kidney injury processes. These metabolites can impact immune responses and metabolic functions through diverse host pathways, resulting in intricate effects on the development of AKI. Notably, SCFAs such as acetate, propionate, and butyrate, which are derived from the fermentation of dietary fibers by the gut microbiota, exhibit potent renoprotective properties 38, 43. For example, sodium butyrate has been observed to mitigate the activation of the nuclear factor-kappa B (NF-κB) signaling pathway, thereby suppressing inflammation and oxidative damage in contrast-induced AKI 44. SCFAs also modulate immune cells. For example, acetate treatment ameliorates sepsis-induced AKI by inhibiting NADPH-oxidase signaling in T cells 45. SCFAs ameliorate AKI by regulating macrophage and neutrophil activation, thereby reducing the production of inflammatory cytokines such as IL-1β, IL-6, and CCL2 19. In I/R-induced AKI, SCFAs have been demonstrated to inhibit leukocyte migration into injured tissues and reduce inflammation 46. Additionally, SCFAs participate in metabolic regulation, as evidenced in sepsis-induced AKI where acetate alleviates kidney oxidative stress and inflammation by activating the AMPK/SIRT1/PGC-1α pathway, consequently contributing to the preservation of kidney function 47. In the gut, SCFAs contribute to the maintenance of gut barrier integrity 48. In summary, changes in SCFA levels caused by gut microbiota dysbiosis may lead to the onset of new AKIs or the exacerbation of existing AKIs.

In addition to SCFAs, amino acid metabolites have been investigated as critical regulators of AKI pathogenesis. Uremic toxins, such as IS and PCS, are classic examples of highly toxic compounds that accumulate during AKI 49. For example, increased IS levels in the serum of patients with AKI correlate with higher mortality and poor prognosis 50. IS enhances IL-1β-induced E-selectin expression through the ROS/MAPKs/NF-κB/AP-1 pathway, thereby exacerbating the progression of AKI 51. Similarly, high PCS levels are associated with kidney function damage in patients with AKI. During AKI, PCS exacerbates kidney injury by inducing inflammation and oxidative stress, thereby manifesting cytotoxic effects 52, 53. However, not all amino acid metabolites are harmful to human health. Another tryptophan metabolite, indole-3-propionic acid (IPA) is known to exhibit protective effects on the kidney. Studies have indicated that IPA protects proximal tubular cells by inhibiting the expression of inflammatory genes 54.

**Figure 2:**
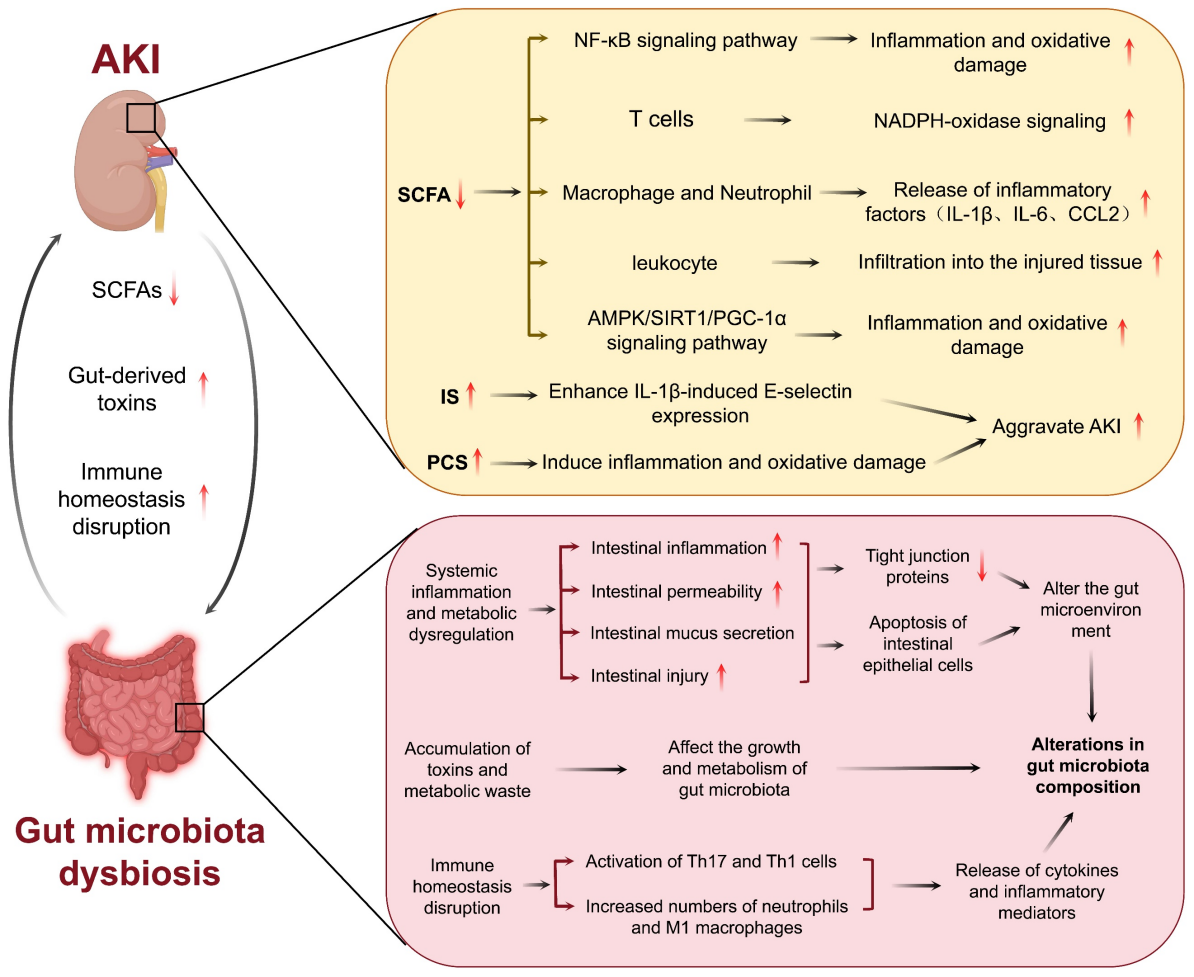
Interaction between gut microbiota and AKI. AKI induces alterations in gut microbiota homeostasis, compromises intestinal barrier function, leading to gut damage. Gut microbiota dysbiosis subsequently exacerbates progression of AKI.

In summary, gut microbiota-derived metabolites are critical for kidney function in patients with AKI. SCFAs can be used to treat AKI by preventing inflammation and oxidative stress-induced kidney injury. In contrast, uremic toxins exert nephrotoxic effects by augmenting inflammation and ROS generation. Thus, the modulation of the gut microbiota and its metabolites may represent a novel treatment strategy for AKI (Figure 2).

### 2.4 Interaction between gut microbiota and CKD

#### 2.4.1 Impact of CKD on gut microbiota

**Figure 3:**
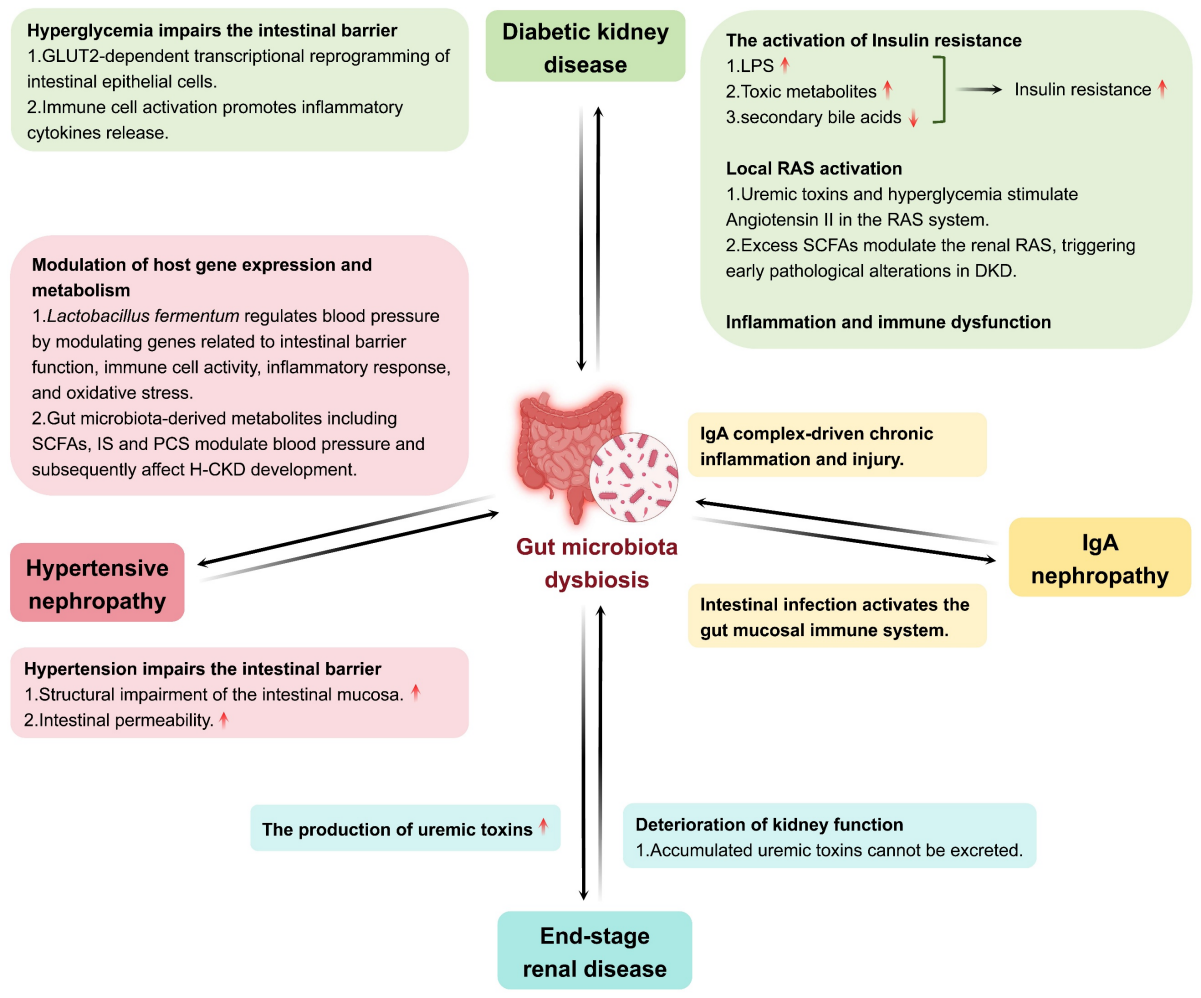
Interaction between gut microbiota and CKD. Gut microbiota dysbiosis engages in a bidirectional pathogenic relationship with multiple forms of CKD, including DKD, lgAN, H-CKD, and ESRD.\

CKD has emerged as a major global public health challenge, creating a significant societal burden. It is characterized by the presence of kidney injury markers or a reduced glomerular filtration rate (GFR) lasting more than three months 55, 56. Over the past three decades, the prevalence and mortality rates of CKD have increased substantially, with forecasts predicting further increases through at least 2029 57. CKD develops progressively, and gut microbiota dysbiosis is increasingly recognized to be involved in the pathophysiology of CKD. The degree of gut microbiota dysbiosis differs in various CKD forms, including DKD, IgA nephropathy (lgAN), hypertensive nephropathy (H-CKD), and end-stage renal disease (ESRD) (Figure 3).

##### Diabetic kidney disease

DKD is the most common cause of CKD, and many studies have shown that the gut microbiota is closely related to DKD 58. Hyperglycemia is a typical symptom of DKD. Multiple studies have indicated that hyperglycemia has an undeniable impact on the intestinal barrier and gut microbiota. First, hyperglycemia drives intestinal barrier permeability through Glut2-dependent transcriptional reprogramming of intestinal epithelial cells, thereby disrupting the integrity of tight and adherens junctions 59. Second, hyperglycemia may promote immune cell hyperactivation in the gut 60. The activated immune cells promote the release of inflammatory cytokines and damage the intestinal barrier. Damage to the intestinal barrier allows for the effects of extraintestinal noxious substances on the gut microbiota. For example, dysbiosis of the gut microbiota occurs in both type 1 diabetes (T1D) and T2D. The gut microbiota composition of patients with T1D has been shown to be different than healthy subjects and the *Firmicutes*/*Bacteroidetes* ratio is shifted to a lower one compared to healthy individuals 61. Another study reported that an increased abundance of *Bacteroides* in patients with T1D was correlated with poor glycemic control 62. Compared to healthy controls, patients with T2D also exhibited lower gut microbiota diversity and richness, similar to that observed in patients with T1D 63, 64. The abundance of butyrate-producing bacteria (*Bifidobacterium*, *Akkermansia*, *Faecalibacterium*) was also lower in patients with T2D 61. Furthermore, a reduction in *Blautia* genus was observed in patients with T2D, which was negatively correlated with glycated hemoglobin and glucose 65. Dysbiosis leads to elevated systemic levels of harmful metabolites, which further promote kidney injury by inducing oxidative stress and activating inflammatory pathways. This not only worsens the inflammatory state of DKD, but may also promote the development of complications. As mentioned previously, hyperglycemia directly affects the gut microbiota and causes a vicious cycle, resulting in the production of more uremic toxins that contribute to the progression of DKD 66.

##### IgA nephropathy

lgAN is an immune-mediated CKD in which IgA immune complexes are deposited in the renal tubular interstitium and mediate chronic inflammation and kidney injury 67. Subsequently, chronic kidney inflammation further aggravates gut microbiota dysbiosis by affecting the intestinal environment through the blood circulation. Patients with lgAN have been observed to experience gut microbiota dysbiosis, known to include decreased SCFA-producing bacteria and increased pro-inflammatory bacteria. SCFAs are required for the preservation of the intestinal barrier function and protection against inflammation. In lgAN, deficits in SCFA-producing bacteria and their fermentation products disrupt this barrier, permitting systemic inflammatory mediators to transcend the circulation, and induce greater kidney inflammation and immune activation 68. Furthermore, the severity of gut microbiota dysbiosis directly correlates with lgAN progression 69, 70. These findings suggest a potential impact of IgAN on the gut microbiota.

##### Hypertensive nephropathy

H-CKD, a type of CKD, is kidney injury caused by long-term hypertension. Kidney injury can cause sodium retention and increase blood volume, which in turn increases blood pressure. Some authors have indicated that alterations in the structure of the gut microbiota are directly related to hypertension. For example, studies show that hypertensive rats display intestinal mucosal damage and detachment of epithelial cells from the mucosal surface 71. Additionally, it has been suggested that hypertension can increase the permeability of the intestinal epithelial barrier 72. This breakdown of intestinal barrier function may be a critical mechanism by which hypertension influences the gut microbiota. The impact of hypertension on the gut microbiota is specifically characterized by a significant reduction in microbial abundance and diversity, with notable enrichment of *Prevotella* and *Klebsiella* accompanied by decreased levels of beneficial bacteria 71. Furthermore, there was no difference in the diversity of gut microbiota between hypertensive patients with and without CKD. However, there are differences between specific bacterial species. In H-CKD, the signature bacteria are *Veillonella parvula* and *Oxalobacter formigenes* and their relative abundance is significantly higher in the H-CKD as compared with hypertension patients. Besides,* Veillonella parvula* and *Oxalobacter formigenes* are potential biomarkers for distinguishing H-CKD patients from those with pure hypertension 71. In metabolism, the level of tauorsodeoxycholic acid was significantly increased in H-CKD patients compared to hypertensive patients without CKD, whereas the levels of other bile acid metabolites did not significantly differ between the two groups 71. These results indicated that H-CKD may affect the gut microbiota.

##### End-stage renal disease

ESRD is a late phase of CKD and is described as a severe and irreversible kidney injury. In patients with ESRD, the retention of uremic toxins damages the intestinal barrier and directly changes the gut microbiota. Gut microbiota dysbiosis is more pronounced in patients with ESRD and mainly reflects the composition and metabolism of the gut microbiota. Some studies have provided evidence that patients with ESRD have low gut microbiota diversity, fewer beneficial bacterial species, and a high prevalence of potential pathogens 73. Dysbiosis of the gut microbiota disrupts immune regulation in various types of cells in the intestine, leading to impaired barrier function and an increase in systemic inflammation, as well as harmful translocation of uremic toxins into the blood circulation 73, 74. Furthermore, kidney dysfunction in patients with ESRD affects gut microbiota metabolism. For example, levels of uremic toxins and secondary bile acids are dramatically increased in the serum metabolome. Uremic toxin precursors such as indole are elevated and SCFAs are significantly lower in the fecal metabolome 75. Interestingly, gut microbiota dysbiosis improves following kidney transplantation, as reflected by a return to normal microbial abundance and diversity, and significant blood microbial uremic toxin reduction 76, 77. This suggests that improving kidney function in patients with ESRD may alleviate gut microbiota dysbiosis.

#### 2.4.2 Involvement of gut microbiota in CKD

Gut microbiota dysbiosis is a crucial factor in CKD progression 78, 79. Metabolic alterations driven by dysbiosis of the gut microbiome are also important in CKD (Table 2). The number of beneficial bacteria decreases, whereas that of pathogenic bacteria increases in patients with CKD 80. Additionally, gut microbiota dysbiosis is known to be linked to the modification of gut barrier function and an increase in intestinal permeability, both of which would contribute to metabolite transport into the systemic circulation, leading to systemic chronic inflammation 81. Persistent chronic inflammation is a secondary injury in the kidney that further accelerates CKD 82. Owing to the diminished kidney function, the elimination of uremic toxins is reduced. These toxins accumulate in the body and trigger the aryl hydrocarbon receptor (AHR) signaling pathway for subsequent injury by promoting oxidative stress and inflammation 83. The following section discusses the function of the gut microbiota and their metabolites in CKD, which is critical for creating gut microbiota-targeted therapy.

##### Diabetic kidney disease

Excessive accumulation of LPS, IS, and PCS in the body may cause insulin resistance. LPS receptors are directly or indirectly involved in inducing insulin resistance 84. It has been shown that gut microbiota dysbiosis can result in an excess of LPS-producing microbiota in the lumen of the gut, promoting systemic low-grade inflammation and enhancing apoptosis of islet cells and insulin resistance in patients with DKD 85, 86. The elevation of toxic metabolites such as PCS and IS can also contribute to insulin resistance 87. In addition, bile acids have also been considered gut microbiota metabolites associated with insulin resistance. The gut microbiota transforms primary bile acids into secondary bile acids, which can modulate glucose metabolism, ameliorate insulin resistance, and improve DKD by binding to their receptors, nuclear farnesoid X receptor (FXR) and membrane-bound Takeda G protein-coupled receptor 5 (TGR5) 87. Gut microbiota dysbiosis leads to the loss of secondary bile acids and suppresses the activation of bile acid receptors FXR and TGR5, contributing to inflammation and insulin resistance 88, 89.

The renin-angiotensin system (RAS) plays an important role in the progression of DKD. Local RAS activation, not circulating RAS, is one of the major triggers of DKD 90. A growing body of evidence has indicated that there is an interaction between gut microbiota and RAS activation 91, 92. The key component of the RAS, Angiotensin II, can be stimulated by uremic toxins and hyperglycemia, leading to renal vasoconstriction, glomerular hyperfiltration, secretion of inflammatory and profibrotic factors, extracellular matrix deposition, and morphological changes in podocytes, thereby promoting the progression of DKD 87, 91, 93. Furthermore, although there is evidence that SCFAs have protective effects in DKD 86, 94, researchers have also pointed out that gut microbiota dysbiosis can lead to excessive SCFA production, particularly of acetate, which may bind to receptors in the kidney and modulate the intrarenal RAS, inducing pathological changes in the early stages of DKD 91, 93, 95.

Although DKD is generally regarded as a non-immune disease, accumulating evidence has suggested that inflammatory responses and the immune system are important for its pathogenesis. The development and progression of DKD are accompanied by inflammation. Chemokines, inflammatory cytokines, and adhesion molecules are involved in several inflammatory pathways that contribute to the complex molecular networks and processes in DKD 96. Several anti-inflammatory treatments can relieve kidney injury in patients with DKD. SCFAs may participate in modulating anti-inflammatory responses by inhibiting histone deacetylase (HDAC) and binding G protein-coupled receptors (GPRs) 97, 98. SCFAs caused reduced levels of inflammatory cytokines, chemokines, and fibrosis-promoting proteins in DKD, leading to improvements in albuminuria, glomerular hypertrophy, podocyte injury, and interstitial fibrosis 86, 94. From the perspective of immunity, dysbiosis of the gut microbiota activates immune cells, resulting in immune dysregulation 91. In addition, the accumulation of gut microbiota-derived metabolites, such as phenyl sulfate (PS), TMAO, IS, and PCS, can continuously stimulate the immune system, potentially leading to the excessive production of inflammatory factors, thereby exacerbating kidney injury in DKD 99.

In summary, gut microbiota dysbiosis is associated with insulin sensitivity, RAS activation, inflammation, and immune system disorders. These factors may contribute separately to the progression toward DKD but may also work synergistically. To better target these mechanisms and prevent DKD progression, future research should expand on the basis of the implicated molecular pathways that regulate gut dysbiosis in DKD, especially those related to metabolism, to derive individualized results.

##### IgA nephropathy

In recent years, it has been emphasized that manipulating the gut microbiota and kidney crosstalk is a novel potential treatment for IgAN 100, 101. IgAN is thought to be initiated by intestinal infections that stimulate the immune system in the intestinal mucosa. Abnormally glycosylated IgA antibodies secreted by the immune cells of the intestinal mucosa are deposited in the kidney 102. The gut microbiota and their metabolites participate in the induction of mucosal immunity in IgAN. These metabolites included IS, PCS, TMAO, and phenylacetylglutamine 69. In an IgAN model, depletion of the gut microbiota using antibiotics resulted in decreased IgA1 mesangial deposition, urinary protein levels, and glomerular inflammation 103, 104. Fresh FMT from healthy donors also significantly reduced proteinuria in two patients with refractory IgAN 105. These findings suggest that the targeted modification of the gut microbiota or its metabolites could be a novel therapeutic option for the treatment of IgAN. Although the modulation of the gut microbiota and its metabolites may constitute a promising therapeutic approach for IgAN, owing to the complexity of the human gut microbiota, it is challenging to identify the gut microbiota or metabolites that are causally associated with IgAN.

*Hypertensive nephropathy* Hypertension is characterized by H-CKD and regulated by the gut microbiota. Gut microbiota has also been reported to be involved in the regulation of hypertension, mainly by acting on host gene expression and metabolite profiles 106. For instance, *Lactobacillus fermentum* alters blood pressure by regulating the genes associated with intestinal barrier function, immune function, inflammation, and oxidative stress 106. Additionally, metabolites from the gut microbiota can control blood pressure, which may influence the progression of H-CKD. SCFAs decrease blood pressure by activating GPR41 and increase it by stimulating Olfr78 72. In addition, higher IS and PCS levels have been associated with increased inflammation and oxidative stress, both of which could be involved in the progression of hypertension and kidney injury 107. However, whether gut microbiota plays a regulatory role in H-CKD requires further investigation.

*End-stage renal disease* Gut microbiota is also involved in the development of ESRD. Gut microbiota is involved in the morbidity and treatment of ESRD. Germ-free mice receiving the gut microbiota of ESRD patients exhibited increases in serum uremic toxins and enhanced kidney fibrosis and oxidative stress, which in turn resulted in further aggravation of kidney injury. The gut microbiota of patients with ESRD is enriched with specific bacterial species, *Eggerthella lenta* and *Fusobacterium nucleatum*, which produce toxins by metabolizing aromatic amino acids 75. Supplementation with probiotics diminishes the abundance of these bacterial groups, decreases toxicity, and enhances kidney function 75. In addition, the gut microbiota can modulate metabolic and immune homeostasis, which can reduce complications in ESRD patients, such as constipation and cardiovascular diseases 73, 108.

Moreover, altered gut microbiota in hemodialysis patients has also been recognized as a potential risk factor for mortality 109, indicating the necessity of recognizing gut microbiota as a novel therapeutic target for the management of ESRD. Dialysis is the treatment modality of choice for patients with ESRD. Emerging evidence has highlighted the association between gut microbiota and dialysis. For instance, the gut microbiota profile of patients undergoing hemodialysis has been reported to be characterized mainly by a dramatic decrease in *Bacteroidetes* and an increase in *Pseudomonas* 74. Various gut microbiota are also found in patients treated with different dialysis therapies such as hemodialysis and peritoneal dialysis 110. In addition, dialysis induces alterations in gut microbiota metabolism, consequently affecting the efficiency of dialysis 111. Together, these findings highlight the critical function of the gut microbiota in the disease progression and therapeutic control of ESRD.

### 2.5 Interaction between gut microbiota and RCC

#### 2.5.1 Impact of RCC on gut microbiota

RCC is a malignant transformation of tubular epithelial cells, and is the most common and fatal urological neoplasm 112. Recently, alterations in the gut microbiota of patients with RCC have been emphasized. Compared to controls, the abundance of *Bacteroides* and *Akkermansia* was significantly increased, and the abundance of *Blautia*, *Bifidobacterium,* and *Megamonas* was significantly decreased in patients with RCC 113. This suggests that RCC may affect the gut microbiota.

#### 2.5.2 Involvement of gut microbiota in RCC

The occurrence and development of RCC are considered to be a multifactorial process, and the gut microbiota may serve as a risk factor for RCC. Previous studies have indicated that the gut microbiota can induce tumorigenesis via several major mechanisms. First, pathogenic microorganisms can directly invade host cells, resulting in cell damage and affecting genome integrity, cell death, and proliferation signaling. These modifications can facilitate the conversion of normal cells into tumor cells 114. Second, pathogenic microorganisms can induce local tissue inflammation by releasing inflammatory molecules such as ROS, reactive nitrogen species (RNS), cytokines, and chemokines secreted by immune cells, which promote tumor growth and metastasis 115. Third, specific microorganisms can prevent the activation of immune cells, resulting in immune dysfunction, allowing the tumor to escape destruction by the host immune system 115. Fourth, pathogenic microorganisms can produce biologically active products or secretions that change the living environment of host cells and destroy the normal biological barrier of host cells, which can also cause tumor development through the host circulatory system away from the microbial growth site 116-118. These findings provide important information concerning the role of gut microbiota in the development and progression of RCC.

In the treatment of RCC, the association of gut microbiota with the immune microenvironment and immunotherapies has been reported 119. For instance, *Akkermansia muciniphila* and *Bacteroides salyersiae* have been shown to activate CD4^+^ and CD8^+^ T cells, reshape the immune microenvironment, and elicit systemic immune responses, thereby enhancing the antitumor efficacy of immunotherapy 120. Furthermore, *Bifidobacterium spp* may enhance the efficacy of PD-1/PD-L1 inhibitors by activating antigen-presenting cells and promoting Th1 immune responses 121. The gut microbiota modulates the immune microenvironment of RCC and the response to immune checkpoint inhibitors (ICIs) largely via its metabolites. SCFAs, common gut microbiota metabolites, influence T cell function, suppress inflammation, and induce tumor cell apoptosis 122. For example, the abundance of *Akkermansia muciniphila* is increased in RCC patients who experience clinical benefit from ICIs therapy 119. This bacterium produces SCFAs, which recruit CD4^+^ T cells and dendritic cells via an IL-12-mediated mechanism, thereby enhancing the efficacy of ICIs in RCC 123. *Firmicutes* also produce SCFAs, which significantly improve treatment responses and thereby enhance clinical outcomes in RCC patients 119, 124. Furthermore, *Bacteroides* can modulate the response to ICIs in RCC patients by inducing a T cell-mediated adaptive immune response through the secretion of capsular polysaccharides 124. Gut microbiota dysbiosis leads to an increase in the tryptophan metabolite kynurenine. Kynurenine binds to and activates the AHR. On one hand, this activation drives epithelial-mesenchymal transition in renal cancer cells, enhancing their migration, invasion, and suppressing apoptosis. On the other hand, sustained AHR activation suppresses anti-tumor immune functions, such as T cell function, thereby promoting tumor immune evasion and metastasis, as well as facilitating the formation of an immunosuppressive microenvironment in RCC 113. In summary, the gut microbiota influences both the immune microenvironment of RCC and the response to ICIs through immune and metabolic mechanisms.

## 3. Strategies targeting gut microbiota

### 3.1 Probiotics, prebiotics and synbiotics

Probiotics have attracted increasing attention as therapeutic agents for gut microbiota dysbiosis and kidney diseases. As described above, the gut barrier deteriorates in AKI, leading to the translocation of metabolites into the systemic circulation, leading to systemic inflammation and kidney dysfunction. Probiotics can modulate gut microbiota, which is helpful in decreasing systemic inflammation and toxin translocation in patients with AKI. Some probiotics may mitigate gut microbiota dysbiosis and inhibit proinflammatory cytokines 19. For instance, the probiotic *Bifidobacterium* has been described as a potential method to mitigate AKI by modulating the gut microbiota, lowering gut inflammation, and improving the intestinal barrier 125. In addition, the association between dysbiosis of the gut microbiota and CKD is complex. The inefficient clearance and excessive accumulation of uremic toxins produced by gut microbiota dysbiosis are pathogenic factors in CKD progression 126, 127. Patients with CKD have been reported to have gut microbiota dysbiosis, which is manifested by a low level of beneficial bacteria and a high level of pathogenic bacteria. Probiotic bacteria can manipulate gut microbiota and suppress the generation of uremic toxins. Probiotics, such as *Lactobacillus acidophilus*, *Bifidobacterium bifidum*, and *Bifidobacterium longum*, have been found to improve kidney function, reduce the concentration of uremic toxins, increasing the quality of life in patients with CKD 128. In addition, probiotics may reduce gut inflammation and regulate other complications associated with CKD 129. Although these studies demonstrated the potential benefits of probiotics, more clinical trials are required to confirm long-term protection against kidney disease and to determine active therapeutic protocols.

Indigestible fibers, which belong to the category of prebiotics, have gained considerable interest with regard to the management of kidney diseases. It has been demonstrated that oligofructose-enriched inulin is a prebiotic that elevates the production of SCFA in the circulation and modulates the level of serum inflammation associated with the modification of the composition of the gut microbiota in CKD rats 130. They may also reduce the concentration of uremic toxins and the activity of proinflammatory cytokines, leading to significant enhancement of kidney function and retardation of CKD progression 131. In addition, clinical trials have reported that inulin-type prebiotics reduce serum uric acid levels in patients with ESRD via the extrarenal pathway, including elevated uric acid degradation and increased purine-degrading species in the gut 132. Other studies have shown increased abundance of beneficial gut bacteria and a decreased abundance of pathogenic bacteria following oligofructose-enriched inulin supplementation, likely resulting in reduced gut-derived toxin levels and improved metabolic profiles in patients with CKD 133. These studies emphasize the therapeutic effect of prebiotics against kidney diseases, which is partly mediated by modifying the gut microenvironment for the proliferation of beneficial bacteria.

Furthermore, synbiotics have great potential for modification of gut microbiota and affect host health. Synbiotics have the advantages of both probiotics and prebiotics to better colonize and function as beneficial bacteria. They are effective against metabolic diseases and chronic inflammation and enhance general gastrointestinal health. For instance, synbiotic therapy, combining high-molecular-weight inulin, fructo-oligosaccharides, and galacto-oligosaccharides as prebiotics with nine probiotic strains from *Lactobacillus*, *Bifidobacteria*, and *Streptococcus genera*, effectively reduces serum levels of IS and PCS in patients with CKD by restoring the gut microbiota composition 134. Other reports have also suggested that long-term use of synbiotics increases the abundance of beneficial bacteria, prevents kidney injury, and reduces serum inflammatory markers 135. Nonetheless, synbiotics face many hurdles in their clinical application. This is an intricate process underlying the matching of probiotics with prebiotics, as using different strains tends to produce diverse effects based on their individual attributes and disease states. For example, although synbiotics have been shown to improve gut microbiota diversity and stability in several clinical trials, they may have negative effects on body weight and metabolism 136. Moreover, the viability of probiotics, the efficacy of prebiotics, and their stability within the gut are critical factors determining the effectiveness of synbiotics. Clinical trials have shown that probiotics are highly susceptible to gastric acid and bile as they migrate through the gastrointestinal tract. Thus, there is an urgent need to design stable carriers for the formulation of synbiotic products 137.

### 3.2 Fecal microbiota transplantation

#### 3.2.1 Application of FMT in kidney disease

FMT, an innovative therapeutic approach, aims to rebalance the gut microbiota by transferring healthy donor microbes and has been demonstrated to have remarkable potential for the treatment of a wide range of diseases 138-140. With the development of research related to the application of FMT in kidney diseases, especially AKI, CKD, and ESRD, several positive findings have emerged. The reconstitution of healthy gut microbiota may be an attractive therapeutic opportunity to either mitigate the progression of microbiota-related diseases or restore kidney health 141. FMT has been shown to help decrease intestinal permeability, repair gut barrier function, and reduce systemic inflammation in AKI 142. Furthermore, FMT alleviates kidney inflammation in CKD by enhancing the production of SCFAs 23. In another CKD model study, FMT enriched the gut microbiota diversity, reduced uremic toxin levels, and improved kidney function 143. Clinical trials in patients with CKD have also shown that the levels of uremic toxins are reduced after 77. In patients with ESRD, immunosuppressive treatments and antibiotics disrupt the gut microbiota, thereby increasing the risk of infection and transplant rejection 144. FMT may reduce the risk of transplant rejection in patients with ESRD by restoring gut microbial balance and attenuating systemic inflammation 145. FMT is a promising new therapy for establishing normal gut microbiota in patients with kidney disease. It has potential uses in AKI, CKD, and ESRD. However, current studies primarily use animal models and there have been few clinical trials exploring its applications. Therefore, further confirmation through large-scale clinical studies is required.

#### 3.2.2 Feasibility and ethical challenges of FMT

The use of FMT for the treatment of kidney diseases is highly feasible. FMT not only plays a role in ameliorating kidney function by modulating the gut microbiota but is also beneficial for the regulation of body metabolism and immunity. However, the safety and efficacy of FMT must be confirmed using standardized donor screening and transplantation procedures. Under strict selection of healthy donors and standardized procedures, FMT may provide a novel therapy for kidney diseases 23.

Despite the therapeutic potential of FMT, its implementation remains fraught with multiple ethical issues. FMT requires the extensive and careful screening of donors to avoid passing on infection and diseases. Nevertheless, there may still be defects in the current screening protocols, and infections may not be completely prevented even after screening 141. Donor screening is also associated with personal privacy issues, for which a high level of protection of donor information is critical. Furthermore, the safety of FMT has not been extensively confirmed in patients with kidney disease. The affected immune function in patients on immunosuppressive therapy might make such patients more vulnerable to infections post-FMT, resulting instead in an aggravation of the original disease. This risk is particularly pronounced in patients with ESRD on immunosuppressive regimens, who may face a substantially higher risk of FMT-related infections compared with other patient populations. ESRD is characterized by chronic uremia, which itself causes profound immune dysregulation 146, 147. Additionally, many ESRD patients are treated with corticosteroids, calcineurin inhibitors, or other immunosuppressive agents, further compromising their immune defenses 148. These individuals often present with multiple comorbidities such as diabetes and cardiovascular disease, which independently increase the risk of infection 149, 150. In ESRD patients, impaired intestinal barrier integrity 146 and immunosuppression create a condition that favors bacterial translocation 151, 152. In this context, even commensal or low-pathogenicity microorganisms from FMT could breach the compromised barrier, causing bacteremia or invasive sepsis. Thus, in patients undergoing immunosuppression, such as those with ESRD, extra caution is required when administering FMT. Further studies are needed before the clinical application of FMT. Finally, another ethical concern arising from the use of FMT is informed consent. Effective FMT is still under investigation; thus, patients must be counseled on potential risks 23. Such challenges may eventually hinder the application of FMT as a potential treatment for kidney disease, unless mitigation strategies are established at the outset. These strategies should range from rigorous research on long-term safety and efficacy to stringent regulatory oversight.

### 3.3 Antibiotics and adsorbents

Gut-targeted drugs, such as antibiotics and adsorbents, are gut-modulating agents and potential drugs for kidney function. For example, in pediatric patients with sepsis-induced AKI, the concomitant use of metronidazole and sulbactam decreased AKI biomarker levels 153. However, they target healthy gut microbiota, not only facilitating the emergence of antibiotic resistance but also inducing immune homeostatic disruption. Additionally, the therapeutic effect of drugs is influenced by the modulation of the gut microbiota by antibiotics. In patients with kidney cancer, a history of antibiotic use is associated with reduced benefit of immunotherapy 121. Furthermore, the adsorbent may interact with the gut microbiota metabolites and prevent their absorption into the circulatory system. Some adsorbents, such as AST-120, may contribute to the protection of the gut barrier, suppression of intestinal permeability to toxins, and reduction of kidney burden through manipulation of the gut microbiota in CKD 154. In summary, gut-directed therapies constitute new avenues for the treatment of kidney disease, particularly those aimed at decreasing gut-derived uremic toxins and remodeling the gut microbiota.

### 3.4 Dietary interventions

Feeding a specialized low-protein and high-fiber diet can beneficially influence gut microbiota profiles and contribute to kidney health. Rebalancing the gut microbiota with low-protein diets has been reported to stimulate the proliferation of beneficial bacteria, such as *Lactobacillaceae* and *Bacteroidaceae* 155. Furthermore, research has shown that low-protein diets decrease the intake of proteins and may decrease the production of IS and PCS 156. SCFAs are produced by the fermentation of dietary fiber in the colon and are crucial for slowing the progression of DKD 157. A diet abundant in dietary fiber can theoretically enhance the production of SCFA-producing bacteria. Subsequently, the SCFAs generated by these fermentative bacteria interact with their receptors, thereby mitigating kidney inflammation and fibrosis in CKD 158, 159. Moreover, SCFAs can promote Treg cell differentiation, which may modulate kidney inflammation 160.

**Figure 4:**
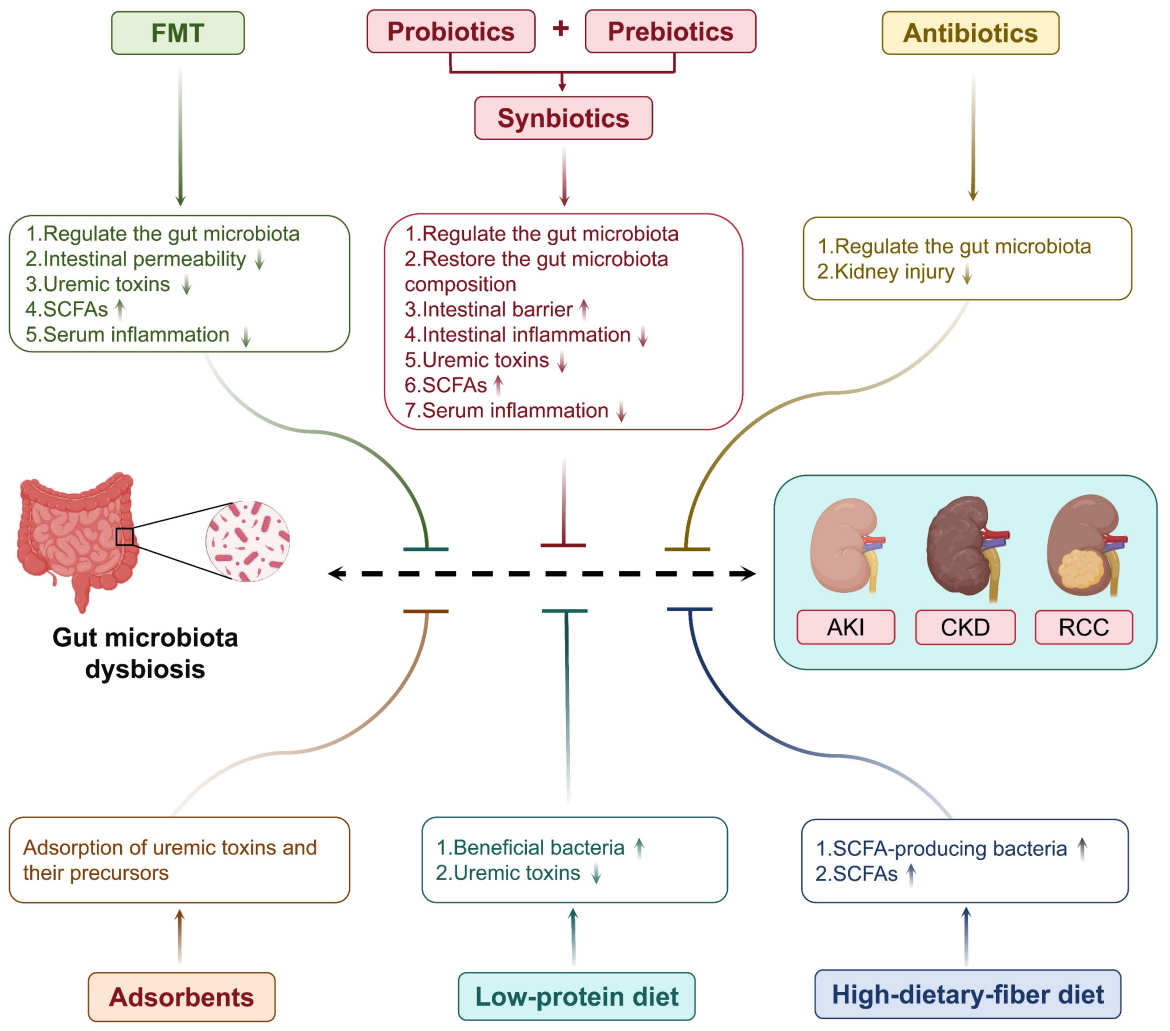
Targeting gut microbiota as a therapeutic strategy for kidney diseases. Multiple gut microbiota-targeted therapeutic approaches can be employed to treat kidney diseases, including probiotics, prebiotics, synbiotics, FMT, antibiotics, adsorbents, and dietary interventions.

In summary, uremic toxin levels could be reduced using low-protein and high-fiber diets, potentially mediated by the modulation of gut microbiota metabolic pathways, which could ultimately benefit the metabolic and anti-inflammatory functions of the kidney. Furthermore, apart from their potential kidney protective effects, these diets offer an effective non-pharmacological therapeutic modality for patients (Table 3 and Figure 4).

### 3.5 The dual role of SCFAs in kidney disease

Targeting the gut microbiota to modulate their metabolites represents a promising therapeutic strategy. Although multiple studies have confirmed that increasing SCFA levels through microbiota modulation can ameliorate kidney disease, SCFAs do not always exert therapeutic effects. On the contrary, under specific concentrations and pathological conditions, they may exhibit detrimental effects. In immune regulation, high concentrations of butyrate not only fail to suppress TNF-α but also promote the expression of IL-1β while inhibiting the anti-inflammatory cytokine IL-10, ultimately demonstrating a pro-inflammatory effect. It can also induce macrophage death, which exacerbates the inflammation 161. Furthermore, low concentrations of butyrate help enhance intestinal barrier integrity, whereas high concentrations may disrupt the barrier structure by inducing epithelial cell apoptosis, promoting immune cell migration, and triggering local inflammation 162. These findings suggest that SCFAs may have an "unfriendly" aspect in the context of kidney diseases. Some studies have pointed out that long-term exposure to SCFA concentrations above physiological levels can impair kidney structure and function. The underlying mechanism involves SCFAs-induced mTOR activation, which promotes the infiltration of inflammatory T cells (Th1/Th17) into kidney tissues. This initiates a cascade of chronic inflammation, fibrosis, and cellular proliferation, culminating in obstructive uropathy and kidney injury 163. In DKD, elevated acetate activates the RAS, inducing glomerular hypertension, proteinuria and tubulointerstitial injury, which accelerates CKD progression. SCFAs may also synergize with other uremic toxins to collectively promote kidney inflammation and fibrosis 91, 93. Furthermore, acetate can promote the expression of proteins involved in cholesterol synthesis and uptake in renal tubular epithelial cells, leading to lipid accumulation and interstitial fibrosis 95.

In summary, SCFAs exert a complex dual role in kidney disease, with outcomes dictated by concentration and pathological context. At appropriate levels, they help maintain the intestinal barrier and modulate immune function. Conversely, elevated concentrations or specific disease states can shift their function toward promoting kidney injury via pro-inflammatory, pro-fibrotic and lipid-dysregulating pathways. Future interventions targeting SCFAs must carefully consider their dose-response relationships and systemic physiological context to avoid potential adverse effects.

## 4. Personalized medicine, challenges and future research

### 4.1 Personalized medicine

Personalized gut microbiota detection and regulation have attracted considerable attention. According to previous studies, variations in the composition and functions of the gut microbiota exist among individuals due to differences in genetics, diet, and age, consequently causing different therapeutic responses 164. Accordingly, regulation of the gut microbiota can provide personalized treatment for patients. For example, differences in the gut microbiota among cancer patients can determine drug activity and adverse effects of targeted therapy 165, and modulation of the microbiota may enhance therapeutic efficacy and reduce side effects 166. Similarly, kidney disease also has considerable potential for individualized detection and regulation of the gut microbiota. Some gut microbes have been identified as major contributors to kidney disease. For example, enhanced *Enterobacteriaceae* and *Bacteroides* are significantly associated with a decline in kidney function, whereas a reduction in beneficial bacteria such as *Lactobacillus* is correlated with the loss of kidney-protective actions 77, 167. Furthermore, the gut microbiota is associated with rejection responses in kidney transplant patients 168. Thus, tailored gut microbiota composition analysis may not only be used as a promising early diagnostic biomarker but also as a foundation for defining personalized therapeutic approaches.

### 4.2 Challenges in clinical application

However, the clinical application of the gut microbiota in kidney disease still faces some challenges. Gut microbiota shows differences between individuals, which makes development of personalized treatment strategies challenging, which is the first problem. In addition, differences in the strains and dosages of probiotics used in studies make the results of clinical trials irreproducible, which complicates the creation of standardized treatment guidelines 169. More importantly, the long-term safety of gut microbiota regulatory strategies remains to be fully established. This issue is of paramount concern for patients with severe disease or immunosuppression. Individuals with uremia or on immunosuppressive therapy often have impaired immunity, increasing their vulnerability to infections linked to FMT. First, despite rigorous donor screening, the risk of transmitting undetectable pathogens cannot be entirely eliminated. Such pathogens, while potentially harmless to healthy individuals, can cause severe and persistent infections in these patients. Secondly, the FMT procedure itself may induce bacterial translocation, increasing the risk of bacteremia and sepsis, especially in patients who already have intestinal wall edema and compromised barrier function. More insidiously, there is a risk of colonization by multidrug-resistant organisms. If undetected resistant bacteria from a donor establish a long-term reservoir in the recipient's gut, it could compromise future antibiotic treatments for subsequent infections, creating a persistent latent health risk. Finally, for immunocompromised patients, FMT poses a unique long-term immune risk by introducing donor immune cells that may launch a delayed, potentially fatal attack on host. To mitigate these risks, rigorous donor selection, proactive surveillance, and long-term follow-up are essential. In conclusion, despite its promise for treating kidney disease, gut microbiota modulation requires solutions to key challenges in standardization and safety. The development of standardized guidelines and rigorous clinical trials to confirm efficacy and safety is essential next steps before this therapy can be widely adopted.

### 4.3 Future research

Emerging evidence suggests that gut microbiota characteristics can predict treatment response in kidney disease patients, going beyond their role as diagnostic biomarkers. For instance, gut microbiota dysbiosis in CKD patients is correlated with disease severity, and gut microbiota characteristics show potential as prognostic biomarkers 66. Research on IgAN provides strong supporting evidence. An analysis of fecal microbiota from 55 IgAN patients revealed a significant enrichment of *Pseudomonas* in non-responders, which was closely associated with poor treatment outcomes 170. Moreover, in a recent prospective study of 69 patients with metastatic RCC treated with nivolumab, a higher response rate was linked to increased levels of *Bacteroides salyersiae*, *Akkermansia muciniphila*, and *Eubacterium siraeum*, along with a lower abundance of *Clostridium clostridioforme* and *C. hathewayi* in the fecal microbiota 171. In summary, the characteristics of the gut microbiota show promise in predicting responses to specific therapies in patients with kidney diseases.

Beyond bacteria, fungi significantly contribute to the microbiota-gut-kidney axis. Despite constituting a minor fraction of the gut microbiota, they regulate gut microecology through antagonistic or synergistic interactions with bacteria, which is essential for maintaining gut microbiota homeostasis 172. A key mechanism of their impact on host health is through immune modulation 173, 174. Fungi such as *Candida*, *Saccharomyces*, and *Aspergillus* can impair the intestinal barrier, allowing components like β-glucan to enter circulation. This promotes systemic inflammation and aggravates kidney injury in CKD 175. Conversely, *Paecilomyces cicadae* can improve DKD in mice by modulating the gut microbiota via the fermentation of astragalus 176, 177. Collectively, fungi play multiple roles in the regulation of kidney health. As another common gut microbe, viruses can infect bacteria to regulate the composition and metabolism of the gut microbiota. This process indirectly influences uremic toxin production and immune homeostasis, which is particularly relevant in CKD patients 178. Furthermore, some viruses carry auxiliary metabolic genes that alter gut microbiota metabolism, including bile acid production, thereby modulating host immunity and triggering systemic inflammation in CKD 178. Enterovirus infection significantly increases the risk of nephrotic syndrome in children. Notably, *Coxsackievirus* may induce the disease by either modulating host immunity or directly damaging glomerular cells 179. Although research on fungi and viruses in the microbiota-gut-kidney axis is still nascent, they may emerge as novel targets for the diagnosis and intervention of kidney diseases in the future.

## 5. Conclusion

The function of the microbiota-gut-kidney axis has attracted increasing attention in basic research and clinical practice. Kidney health is related to the gut microbiota, which can affect kidney physiology through the immune and inflammatory pathways. Gut microbiota dysbiosis is a manifestation of kidney disease and is one of the key regulators of its development. Modulation of gut microbiota may provide promising therapeutic opportunities for kidney diseases. Gut microbiota-targeted therapies such as probiotics, prebiotics, synbiotics, and FMT can restore the gut microbiota and ameliorate both inflammation and gut microbiota-derived uremic toxins. Research on individualized treatment strategies is ongoing. Precise assessment of the gut microbiota of each patient allows the development of tailored approaches to modulate it in order to enhance therapeutic efficacy. Although these results are encouraging, more clinical evidence is required to validate the efficacy and safety of these strategies, which are widely accepted as standard treatments. In summary, the modulation of the gut microbiota is a promising therapeutic strategy for treating kidney diseases. However, before these techniques can be safely and effectively introduced into the clinic, issues regarding standardization and ethics must be addressed in future studies.

## Figures and Tables

**Table 1:** The role and mechanisms of gut microbiota-derived metabolites in AKI.

Model	Metabolite	Function	Mechanism	Reference
Contrast-induced AKI	SCFAs	Protective	Sodium butyrate reduces inflammation and oxidative stress through suppression of NF-κB pathway activation.	44
Sepsis-induced AKI	SCFAs	Protective	Acetate ameliorates AKI by suppressing NADPH oxidase signaling in T cells.	45
I/R-induced AKI	SCFAs	Protective	SCFAs inhibit the release of inflammatory cytokines by modulating macrophage and neutrophil activity, ultimately ameliorating AKI.	19
I/R-induced AKI	SCFAs	Protective	SCFAs reduce leukocyte infiltration into injured tissues and attenuate kidney inflammation.	46
Sepsis-induced AKI	SCFAs	Protective	Acetate ameliorates oxidative stress and inflammation by activating the AMPK/SIRT1/PGC-1α axis.	47
I/R-induced AKI	IS	Pathogenic	IS exacerbates the progression of AKI through the ROS/MAPKs/NFκB/AP-1 pathway.	51
I/R-induced AKI	PCS	Pathogenic	PCS promotes kidney injury by inducing inflammation and oxidative stress.	52, 53

AKI, acute kidney injury; IS, indoxyl sulfate; PCS, p-cresol sulfate; SCFAs, short-chain fatty acids.

**Table 2:** The role and mechanisms of gut microbiota-derived metabolites in CKD.

Model	Metabolite	Function	Mechanism	Reference
DKD	LPS	Pathogenic	LPS promotes systemic inflammation in DKD patients and facilitates pancreatic islet cell apoptosis and insulin resistance.	85, 86
DKD	IS and PCS	Pathogenic	IS and PCS induce insulin resistance.	87
DKD	Secondary bile acids	Protective	Secondary bile acids alleviate inflammation and insulin resistance through FXR/TGR5 activation.	88, 89
DKD	IS and PCS	Pathogenic	Uremic toxins activate Angiotensin II, leading to kidney vasoconstriction, glomerular hyperfiltration, secretion of inflammatory and profibrotic cytokines, extracellular matrix deposition, and podocyte morphological changes, thereby promoting the progression of DKD.	87, 91, 93
DKD	SCFAs	Protective	SCFAs reduce the levels of inflammatory cytokines, chemokines, and fibrosis-promoting proteins in DKD.	86, 94
DKD	Excessive SCFAs	Pathogenic	Excessive SCFAs modulate the intrarenal RAS, triggering early pathological changes in DKD.	91, 93, 95
DKD	TMAO, IS, PCS, and PS	Pathogenic	Activates the immune system, resulting in the overproduction of inflammatory cytokines.	99
IgAN	IS, PCS, TMAO, and phenylacetylglutamine	Pathogenic	Stimulates the intestinal mucosal immune system.	69
H-CKD	SCFAs	Protective	SCFAs decrease blood pressure by activating GPR41.	72
H-CKD	SCFAs	Pathogenic	SCFAs increase blood pressure by activating Olfr78.	72
H-CKD	IS and PCS	Pathogenic	IS and PCS increase inflammation and oxidative stress.	107
ESRD	IS, PCS, and TMAO	Pathogenic	Exacerbates kidney fibrosis and oxidative stress.	75

lgAN, IgA nephropathy; H-CKD, hypertensive nephropathy; DKD, diabetic kidney disease; ESRD, end-stage renal disease; IS, indoxyl sulfate, PCS, p-cresol sulfate; SCFAs, short chain fatty acids; TMAO, trimethylamine N-oxide.

**Table 3:** Therapeutic strategies targeting gut microbiota.

Strategies	Diseases	Application examples	Reference
Probiotics	AKI	*Bifidobacterium* mitigates AKI through regulation of the gut microbiota, inhibition of intestinal inflammation, and restoration of the intestinal barrier.	125
Probiotics	CKD	Probiotic supplementation with *Lactobacillus acidophilus*, *Bifidobacterium bifidum*, and *Bifidobacterium longum* improves kidney function and reduces serum uremic toxin levels in CKD patients.	128
Prebiotics	CKD	Oligofructose-enriched inulin can increase circulating SCFA levels, modulate serum inflammation by altering the composition of the gut microbiota.	130
Prebiotics	ESRD	Inulin-type prebiotics reduced serum uric acid levels in ESRD patients by modulating the gut microbiota.	132
Prebiotics	CKD	Oligofructose-enriched inulin improves the gut microbiota dysbiosis and its metabolic profile in patients with CKD.	133
Synbiotics	CKD	Synbiotic therapy reduces serum IS and PCS levels and inflammation in patients with CKD by restoring the gut microbiota composition.	134
FMT	AKI	FMT decreases intestinal permeability, repairs gut barrier function, and reduces systemic inflammation in AKI.	142
FMT	CKD	FMT improves kidney function in CKD patients by modulating the gut microbiota, enhancing the production of SCFAs, and reducing uremic toxin levels.	23, 143
FMT	ESRD	FMT restores the balance of gut microbiota and reduces systemic inflammation, potentially lowering the risk of transplant rejection in patients with ESRD.	145
Antibiotics	AKI	The combination of metronidazole and sulbactam can reduce the levels of AKI markers.	153
Adsorbents	CKD	AST-120 is an oral adsorbent that reduces the progression of CKD by adsorbing uremic toxins and their precursors in the gut.	154
Low-protein diets	CKD	A low-protein diet promotes the proliferation of beneficial bacteria, such as *Lactobacillaceae* and *Bacteroidaceae*.	155
Low-protein diets	CKD	A low-protein diet reduces the production of IS and PCS.	156
Dietary fiber	DKD	SCFAs produced by dietary fiber fermentation slow DKD progression.	157
Dietary fiber	CKD	A diet rich in dietary fiber enriches SCFA-producing bacteria, and the SCFAs generated by these bacteria can alleviate kidney inflammation and fibrosis.	158, 159

AKI, acute kidney injury; CKD, chronic kidney disease; DKD, diabetic kidney disease; ESRD, end-stage renal disease; IS, indoxyl sulfate; PCS, p-cresol sulfate; SCFAs, short chain fatty acids.

## Abbreviations

AKI: acute kidney injury
CKD: chronic kidney disease
RCC: renal cell carcinoma
FMT: fecal microbiota transplantation
IS: indoxyl sulfate
PCS: p-cresol sulfate
TMAO: trimethylamine N-oxide
AGEs: Advanced Glycation End Products
AHR: aryl hydrocarbon receptor
ROS: reactive oxygen species
SCFAs: short chain fatty acids
I/R: ischemia/reperfusion
NF-κB: nuclear factor-kappa B
IPA: indole-3-propionic acid
GFR: glomerular filtration rate
DKD: diabetic kidney disease
H-CKD: hypertensive nephropathy
ESRD: end-stage renal disease
T1D: type 1 diabetes
T2D: type 2 diabetes
FXR: farnesoid X receptor
TGR5: Takeda G protein-coupled receptor 5
RAS: renin-angiotensin system
HDAC: histone deacetylase
GPRs: G protein-coupled receptors
PS: phenyl sulfate
RNS: reactive nitrogen species
ICIs: immune checkpoint inhibitors
